# Successful Management of a Delayed Traumatic Hemothorax by Timely Imaging Re-evaluation in a Hybrid Emergency Room System

**DOI:** 10.7759/cureus.94444

**Published:** 2025-10-13

**Authors:** Saya Hao, Tensei Suzuki, Wataru Takayama, Mitsuhiro Kishino, Koji Morishita

**Affiliations:** 1 Department of Acute Critical Care and Disaster Medicine, Institute of Science Tokyo, Tokyo, JPN; 2 Department of Diagnostic Radiology, Institute of Science Tokyo, Tokyo, JPN

**Keywords:** computed tomography, delayed hemothorax, hybrid emergency room system, post-treatment imaging, trauma

## Abstract

Delayed hemothoraces are rare in patients with trauma, and their optimal management has not been well established. We report a well-managed case of delayed hemothorax diagnosed on post-treatment computed tomography (CT) and managed through endovascular intervention in a hybrid emergency room system. A 24-year-old woman presented in shock after falling from a height of 10 meters. With no external signs of trauma, initial whole-body CT revealed an unstable pelvic ring fracture with active extravasation. Following resuscitative endovascular balloon occlusion of the aorta, transarterial embolization was performed for pelvic bleeding 1 h after patient arrival. CT performed immediately after embolization revealed a delayed right hemothorax. Selective angiography revealed a pseudoaneurysm in a lateral upper intercostal artery branch, which was successfully treated via embolization. In cases of severe blunt trauma requiring invasive interventions, post-treatment CT should be considered even in the absence of abnormalities on the initial scan because of the potential for delayed hemothoraces.

## Introduction

A hemothorax is one of the injuries associated with blunt chest trauma, along with pneumothorax and pulmonary contusion [1]. A delayed hemothorax, defined as a condition in which a hemothorax is not observed on the initial examination but is subsequently detected, is a rare entity that accounts for 4.2% to 12.2% of all traumatic hemothoraces [2-4]. It is frequently associated with rib fractures and is known to occur more commonly in older adult patients [5,6]. Hemothoraces missed during the initial evaluation may progress insidiously, with small collections on the initial computed tomography (CT) scan often managed conservatively, whereas larger delayed collections can lead to respiratory failure or hemorrhagic shock and frequently require drainage or surgical intervention [7,8]. In reported cases, delayed hemothoraces have led to hemodynamic collapse requiring thoracotomy or complications such as empyema, fibrothorax, and prolonged ventilation, emphasizing that delayed recognition can increase mortality and the need for invasive management [2,6,9,10]. Management strategies for delayed hemothoraces range from close observation and tube thoracostomy to endovascular or surgical interventions, depending on the hemodynamic status and the underlying source of bleeding [9].

While close observation is sometimes chosen, the timing of delayed hemothorax onset has no clear definition. Reported intervals vary widely, from as early as two hours to as late as 44 days after trauma [5,6,11]. In this manuscript, we defined a delayed hemothorax as a lesion that was not detected on the initial CT. The optimal timing for repeat imaging also remains unclear, particularly due to concerns about radiation exposure and the risks associated with patient transfer.

To address this diagnostic challenge, we report a unique case of a delayed hemothorax detected on post-treatment CT and effectively managed through endovascular intervention within a hybrid emergency room system (HERS). A HERS allows integrated trauma management, including resuscitation, imaging, surgery, and endovascular interventions, without requiring patient transfer, potentially enabling timely diagnosis and treatment in critically ill patients [12].

In this manuscript, we demonstrate the importance of considering delayed hemothoraces even when no abnormalities are detected on an initial CT, and we emphasize that the timing of imaging re-evaluation is clinically crucial in patients with severe trauma.

## Case presentation

A 24-year-old woman with no significant medical history was brought to our emergency department after a suicide attempt, during which she fell from an estimated height of 10 m and landed on a concrete surface. On the arrival of emergency medical services, she was in shock with a systolic blood pressure of 60 mmHg and a pulse rate of 140 b/min. The patient was transferred to our hospital for management in the HERS.

Upon arrival at our emergency department, her vital signs were as follows: oxygen saturation of 100% while receiving 10 L/min oxygen via a reservoir mask; pulse rate ranging from 140 to 150 b/min; blood pressure, 85/56 mmHg; respiratory rate, 20 breaths/minute; and temperature, 35.9°C. Her Glasgow Coma Scale score was E4 V4 M6 with no neurological deficits. Her pupils were 4 mm/4 mm and reactive to light. The patient’s vital signs and laboratory findings on admission are summarized in Table 1.

**Table 1:** Vital signs and laboratory findings on admission SpO₂: oxygen saturation; PR: pulse rate; BP: blood pressure; RR: respiratory rate; BT: body temperature; GCS: Glasgow Coma Scale; WBC: white blood cell count; RBC: red blood cell count; Hb: hemoglobin; PLT: platelet count; PT: prothrombin time; INR: international normalized ratio; APTT: activated partial thromboplastin time; Fbg: fibrinogen; CRP: C-reactive protein; AST: aspartate aminotransferase; ALT: alanine aminotransferase; LDH: lactate dehydrogenase; CRE: creatinine; eGFR: estimated glomerular filtration rate; BUN: blood urea nitrogen.

Parameter	Result	Reference range	Measurement unit
SpO₂	100% (10 L/min O₂ via reservoir mask)	≥95% (room air)	%
PR	140–150	60–100	beats/min
BP	85/56	120/80	mmHg
RR	20	12–20	breaths/min
BT	35.9	36.0–37.5	°C
GCS	E4 V4 M6	15 (normal)	–
Pupils	4 mm/4 mm, reactive	–	–
WBC	17.9	4.0–10.0 ×10³/µL	×10³/µL
RBC	329	380–480 ×10⁴/µL	×10⁴/µL
Hb	10.1	13.0–17.0	g/dL
PLT	23.0	150–400	×10³/µL
PT	14.9	10–13	sec
PT-INR	1.27	0.9–1.1	–
APTT	31.1	25–35	sec
Fbg	152	200–400	mg/dL
D-dimer	177.4	<1.0	µg/mL
CRP	0.03	<0.3	mg/dL
AST	44	10–40	U/L
ALT	20	5–45	U/L
LDH	283	120–240	U/L
CRE	0.89	0.6–1.1	mg/dL
eGFR	58	>60	mL/min/1.73 m²
BUN	12.6	8–20	mg/dL

**Figure 1:**
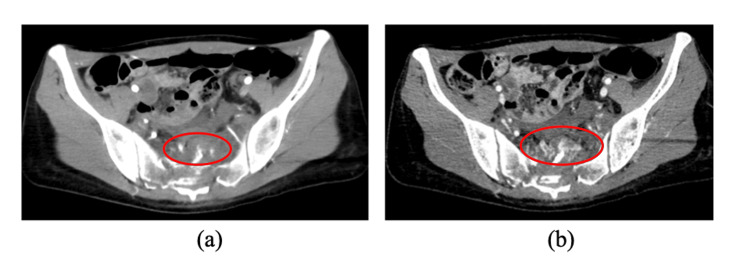
Initial contrast-enhanced computed tomography (a) Arterial phase: Active extravasation of contrast is observed on the anterior surface of the sacrum (red circle). (b) Venous phase: An increase in contrast accumulation is noted (red circle).

Physical examination revealed no apparent chest trauma, but pelvic instability was noted. Following the rapid administration of 1,000 mL of lactated Ringer’s solution, her systolic blood pressure increased to 110 mmHg. Contrast-enhanced CT was performed 15 min after the patient’s arrival at the hospital. Whole-body CT revealed a first lumbar vertebral burst fracture (AO Classification Type A4), unstable sacral fracture (AO Classification Type C3), and active extravasation on the anterior sacral surface (Figure 1). No major vessel injuries or hematomas were identified in the thoracic region.

**Figure 2:**
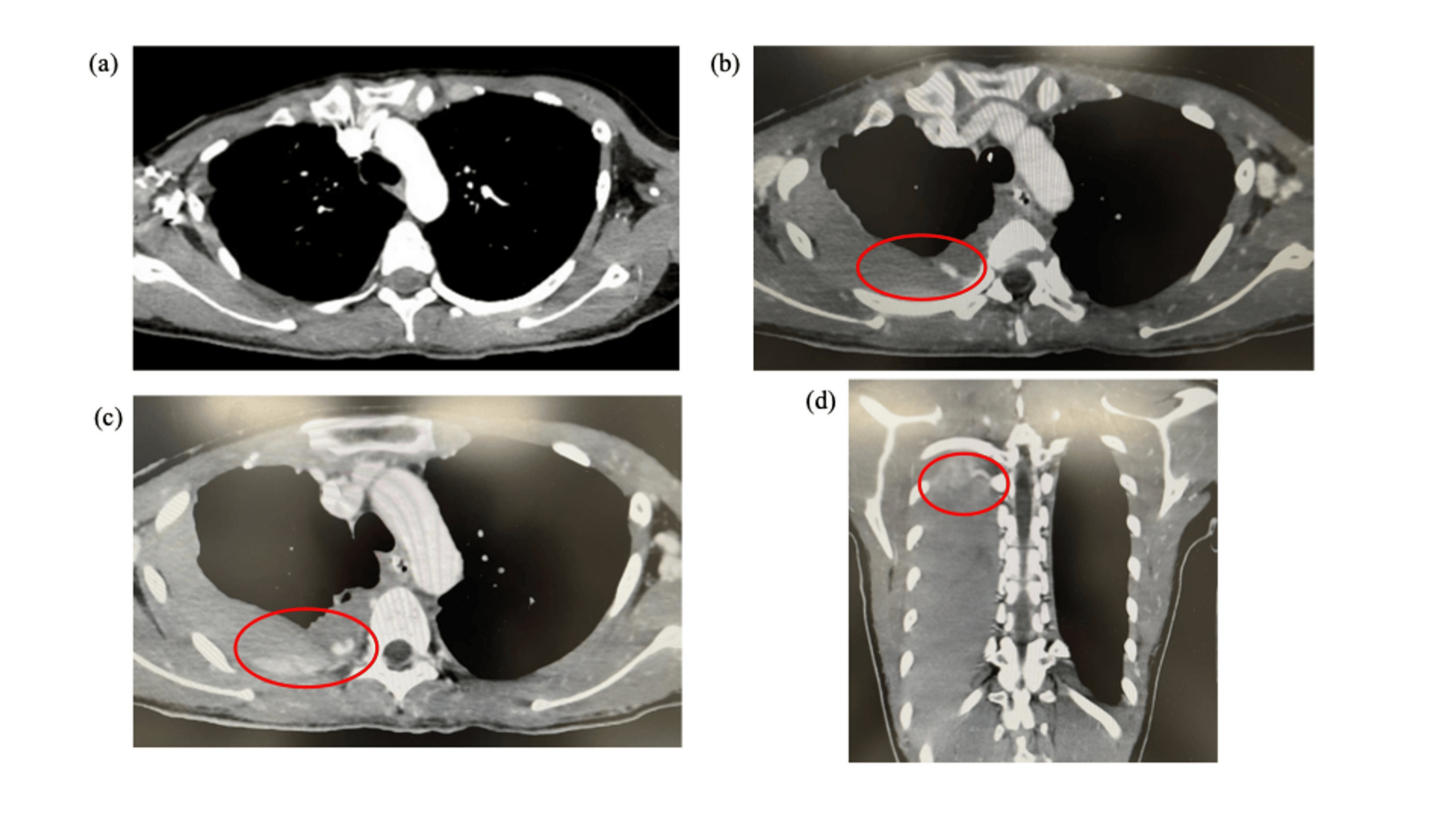
Comparison between the initial and post-treatment computed tomography performed 3 h apart (a) The initial computed tomography revealed no obvious pathological findings (b), (c), (d) Post-treatment computed tomography revealed a massive right-sided hemothorax with active contrast extravasation (red circle) that had not been evident on the initial scan, prompting emergency tube thoracostomy followed by repeat embolization.

**Figure 3:**
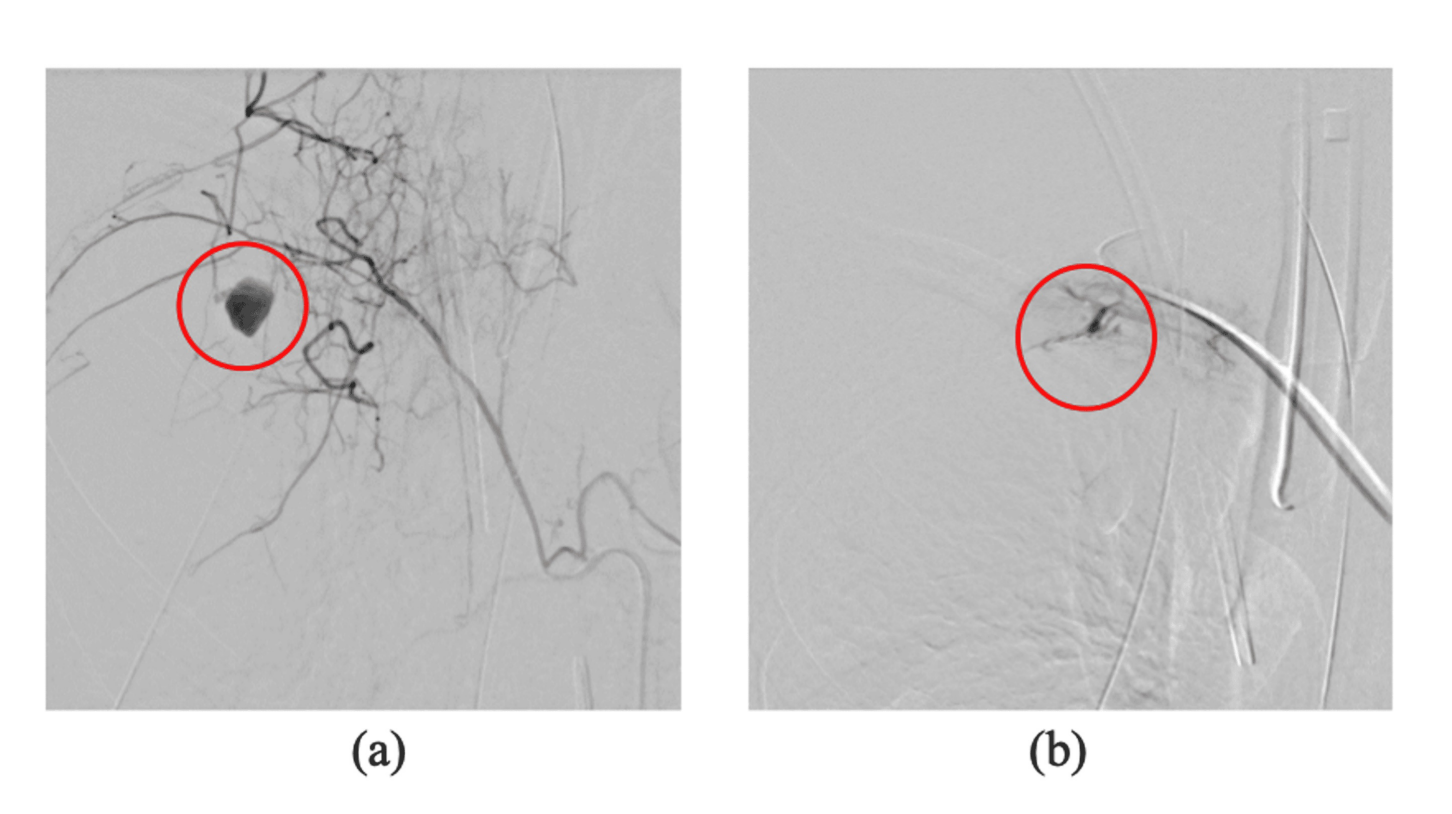
Findings from the second interventional radiology (thoracic interventional radiology) (a) A pseudoaneurysm was identified in the lateral branch of the upper intercostal artery (red circle). (b) Post-embolization: Resolution of extravasation is confirmed.

**Figure 4:**
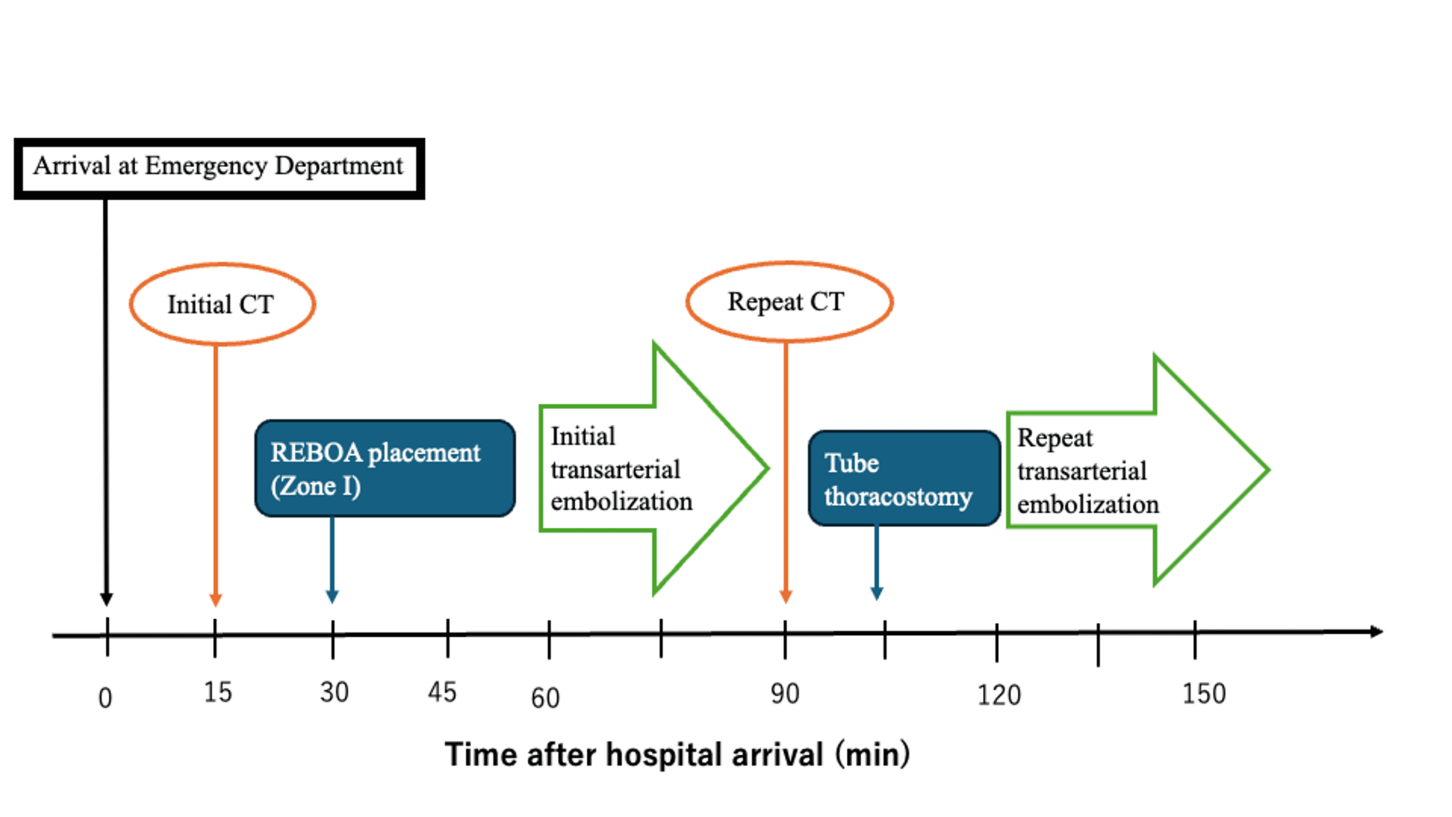
Timeline of the patient’s clinical course REBOA: Resuscitative endovascular balloon occlusion of the aorta

Following contrast-enhanced CT, the patient’s systolic blood pressure dropped to 60 mmHg after the initial fluid resuscitation. Transfusion of red blood cells and fresh frozen plasma was initiated, and resuscitative endovascular balloon occlusion of the aorta (REBOA) was performed with balloon inflation in Zone I, as there were concerns about hemorrhage from other traumatic injuries. These interventions successfully elevated the systolic blood pressure to 120 mmHg. With the REBOA maintained in a partially inflated state, transarterial embolization was performed for pelvic bleeding 1 h after hospital admission. During the procedure, active contrast extravasation was identified from the bilateral sacral and left iliolumbar arteries, and transarterial embolization was performed using gelatin sponge particles. Immediately after the completion of embolization, the REBOA was deflated, and the patient’s blood pressure remained stable. At our institution, which is equipped with a HERS, follow-up CT is selectively performed in patients with polytrauma, particularly after transarterial embolization or surgery, or when laboratory tests suggest progression of anemia or coagulopathies. This approach aims to detect delayed or recurrent bleeding, assess treatment efficacy, and identify missed injuries. A post-treatment CT was obtained in this case according to this protocol. The CT revealed a right-sided hemothorax with active extravasation of contrast into the thoracic cavity (Figure 2). An intercostal drain was inserted into the right thoracic cavity, draining approximately 500 mL of blood. A second transarterial embolization was performed 1 h after the initial procedure. Angiography of the right-sided third, fourth, and fifth intercostal arteries was performed, and a pseudoaneurysm was identified in the lateral perforating branch of the right third intercostal artery. Selective embolization was successfully performed using a gelatin sponge and coils (Figure 3). The chronological course of the patient’s management is shown in Figure 4.

Follow-up CT on days 1 and 5 of hospitalization showed no evidence of rebleeding. On day 6 of hospitalization, posterior spinal fusion surgery (Th12-L2) was performed for the L1 vertebral body fracture. The postoperative course was uneventful, and no procedure-related complications occurred at the site of the angiographic intervention. The patient was extubated on postoperative day 7 and transferred to the psychiatric department on day 54.

## Discussion

We encountered a rare case of a delayed hemothorax that was successfully managed through post-treatment CT performed in a HERS, leading to favorable clinical outcomes. Although delayed hemothoraces are commonly associated with rib fractures [5], this case was unique because it involved upper intercostal artery injury and bleeding in the absence of rib fractures. The intercostal arteries are well recognized as a major source of bleeding in hemothoraces; however, the development of a pseudoaneurysm localized to the lateral perforating branch, as observed in this case, is relatively rare when absent prior to trauma and has been infrequently reported. Nevertheless, several case reports have documented such lesions in patients with blunt trauma-associated hemothoraces, suggesting that this potential source of bleeding should be considered in similar clinical scenarios [13]. 

Furthermore, the absence of abnormal findings on the initial CT may have contributed to the delayed diagnosis, which could have affected the patient’s prognosis. In the present case, the relatively early onset of the delayed hemothorax (within three hours) may have been influenced by the progression of a trauma-induced coagulopathy and the 20-minute REBOA balloon inflation in Zone 1.

Severe trauma may induce coagulopathies secondary to tissue injury, shock, hypothermia, acidosis, or the dilutional effects of massive fluid resuscitation, leading to rebleeding from small vessels that initially achieved hemostasis [14,15]. In addition, recent physiological studies have demonstrated that REBOA markedly elevates proximal arterial pressure and systemic vascular resistance, yielding hemodynamic effects comparable to aortic cross-clamping. As a result, coronary and cerebral perfusion are enhanced, while left ventricular afterload and contractility increase, which may unmask previously subclinical bleeding sources [16,17]. Based on these findings, it is plausible that aortic occlusion by the REBOA elevated the proximal perfusion pressure, thereby unmasking previously contained bleeding in the present case [18,19]. In patients with multiple traumas, post-treatment CT should be considered after vascular interventions such as surgery or transarterial embolization or procedures associated with significant hemodynamic changes due to the risk of progression of other injuries. This recommendation is consistent with existing trauma imaging guidelines; for example, the ACS TQIP Best Practices in the Management of Traumatic Brain Injury (TBI) guidelines state that repeat CT imaging should be considered to identify evolving lesions and guide treatment [20]. While these guidelines are specific to TBIs and guidelines addressing polytrauma or truncal injuries exist only at the level of case series, the underlying principles nonetheless support the use of follow-up CT after major interventions in patients with polytrauma.

This case highlights key management strategies for patients with polytrauma: (i) delayed hemothorax should be considered even in patients without rib fractures or abnormal initial CT findings, and (ii) post-treatment CT should be considered, particularly after interventions that may affect hemodynamic stability or in cases where laboratory tests reveal worsening anemia or coagulopathy [13,20]. However, due to radiation exposure risks, further protocol refinement is required to define the appropriate indications. 

## Conclusions

This case illustrates a rare instance of a delayed hemothorax caused by the rupture of an intercostal artery pseudoaneurysm in the absence of rib fractures, which was promptly diagnosed and managed through post-treatment CT in a HERS. It underscores the importance of maintaining clinical suspicion for delayed hemothoraces even when the initial CT reveals no abnormalities.

Clinicians should consider follow-up CT not only for hemodynamically unstable patients but also for those who remain stable when high-risk injury mechanisms or invasive procedures are involved. In institutions equipped with a HERS, repeat imaging can be performed without transfer, but the benefits of early detection must be weighed against radiation exposure. Further studies are needed to establish evidence-based indications for follow-up CT in severe trauma.
